# Supplementary material for: The Prediction of Gestational Hypertension, Preeclampsia and Fetal Growth Restriction via the First Trimester Screening of Plasma Exosomal C19MC microRNAs
Source: Int J Mol Sci. 2019 Jun 18;20(12):2972. doi: 10.3390/ijms20122972 (PMC6627682; doi:10.3390/ijms20122972)
Supplement: Supplementary file 1 [file ijms-20-02972-s001.pdf]

Table S1. The assessment of the normality of experimental data using Shapiro-Wilk test.

	**Shapiro-Wilk test**	
miR-516b-5p	W = 0.78548	$p < 0.001$
miR-517-5p	W = 0.39129	$p < 0.001$
miR-518b	W = 0.41613	$p < 0.001$
miR-520a-5p	W = 0.22953	$p < 0.001$
miR-520h	W = 0.60976	$p < 0.001$
miR-525-5p	W = 0.19550	$p < 0.001$

Normality of the data was assessed using Shapiro-Wilk test, which indicated that our experimental data did not follow a normal distribution.

Table S2. The presentation of no statistically significant results.

	**miR-516b-5p**	**miR-518b**	**miR-520h**		
GH vs NP	$p = 0.194$	$p = 1.0$	$p = 0.741$		
PE vs NP	$p = 1.0$	$p = 0.735$	$p = 1.0$		
	**miR-516b-5p**	**miR-517-5p**	**miR-518b**	**miR-520h**	**miR-525-5p**
FGR vs NP	$p = 0.840$	$p = 0.937$	$p = 0.278$	$p = 0.282$	$p = 0.633$

No difference in microRNA expression was observed in circulating plasma exosomes within 10 to 13 weeks of gestation in women affected with GH, PE or FGR when the comparison to the controls was performed using non-parametric statistical test (the Kruskal-Wallis test). PE, preeclampsia; GH, gestational hypertension; FGR, fetal growth restriction; NP, normal pregnancies.
